# Systematic Review of Acute Isolated Distal Radioulnar Joint Dislocation: Treatment Options

**DOI:** 10.3390/jcm13247817

**Published:** 2024-12-21

**Authors:** Konstantinos Zampetakis, Ioannis M. Stavrakakis, Kalliopi Alpantaki, Grigorios Kastanis, Ioannis Ktistakis, Alexandros Tsioupros, Nikolaos Ritzakis, Constantinos Chaniotakis

**Affiliations:** Department of Orthopaedics and Trauma Surgery, “Venizeleion” General Hospital of Heraklion, 71409 Crete, Greece; k_zampetakis@yahoo.com (K.Z.); i.m.stavrakakis@gmail.com (I.M.S.); apopaki@yahoo.gr (K.A.); kastanisg@gmail.com (G.K.); giannis.ktistakis@gmail.com (I.K.); alexandros-tsioupros@hotmail.com (A.T.); nikosritz7@gmail.com (N.R.)

**Keywords:** distal radioulnar joint, dislocation, wrist injury, carpal instability

## Abstract

**Background/Objectives**: Acute isolated distal radioulnar joint (DRUJ) dislocations are rare and often misdiagnosed during initial evaluation due to subtle clinical presentation, low index of suspicion, and imaging barriers. Prompt diagnosis and treatment are critical to avoid chronic instability, limited wrist mobility, and osteoarthritis. This systematic review evaluates the functional outcomes of conservative and surgical treatment protocols for acute isolated DRUJ dislocations. **Methods**: A systematic search of PubMed, Scopus, and Mendeley databases (2000–2024) was conducted following PRISMA guidelines. Inclusion criteria involved adult patients with isolated DRUJ dislocations diagnosed and managed within one week of injury. Studies reporting on underage patients, associated fractures, delayed management, and open injuries were excluded. Data on demographics, injury mechanism, diagnostic methods, treatment protocols, and functional outcomes were extracted and analyzed. **Results**: In total, 22 cases across 20 studies were included. The majority (90.9%) were males, with a mean age of 37.9 years (range: 20–70 years). Falls and sports injuries were the major causes, with volar dislocations predominating (18/22). The misdiagnosis rate was equal to 18%. Most cases were treated conservatively with closed reduction and immobilization for an average of 4.9 weeks. Operative treatment was performed in 6 cases, mainly following failed closed reductions. Functional outcomes were generally favorable, although the same parameters were not consistently studied in all patients. Overall, 82% (14 of 17 patients) achieved a full range of motion; 88% (14 of 16 patients) reported no pain, and all assessed cases had stable DRUJs at follow-up. **Conclusions**: This review highlights the rarity and diagnostic challenges of this injury. The functional outcomes of both conservative and operative treatment are generally satisfactory. Conservative treatment should be the first-line approach, with surgery reserved for irreducible or unstable cases. Future research using standardized outcome measures is needed to provide guidance for clinicians.

## 1. Introduction

Acute isolated distal radioulnar joint (DRUJ) dislocations comprise an uncommon injury pattern. Usually, they coexist with fractures of the distal radius and ulnar styloid, or they occur as part of more complex injuries like Essex–Lopresti and Galeazzi fractures [1,2]. The timely recognition of isolated DRUJ dislocations can be challenging, as up to half of the cases are missed during the initial presentation [2,3]. This is mainly attributed to a low index of suspicion, subtle clinical presentation, and failure to obtain proper X-rays [4]. Interestingly, only a small degree of forearm rotation in the lateral X-rays is just enough to mislead diagnosis [5,6]. If this injury is overlooked and left untreated, it can lead to devastating functional outcomes, including secondary DRUJ instability, loss of pronation-supination, painful wrist, and osteoarthritis [2,7,8].

Understanding the complex anatomy of DRUJ is a prerequisite to accurate diagnosis and management. DRUJ is the inherently unstable articulation between the ulnar head and sigmoid notch of the distal radius, which facilitates forearm pronation-supination [9]. The stability of the joint primary relies on the triangular fibrocartilage complex (TFCC) but is also reinforced by other passive stabilizers such as the joint capsule, ulnocarpal ligaments, and the interosseous membrane (IOM) [10]. The pronator quadratus muscle and the extensor carpi ulnaris (ECU) tendon are characterized as active stabilizers of the DRUJ [10,11,12].

DRUJ dislocations are categorized into volar or dorsal. Although the ulna is the stable unit of the forearm around which the radius axially rotates, it is of common acceptance that the direction of DRUJ dislocation is defined by the position of the ulnar head in relation to the distal radius. Dorsal dislocations, as a result of hyperpronation injury, are more common than volar dislocations, which conversely occur with hypersupination injury [2,6,13,14].

Historically, the first documented acute isolated DRUJ dislocation was described by Pierre-Joseph Desault in 1777 regarding a cadaver case; yet, to this date there are limited comprehensive studies to elucidate this rare clinical entity [15,16]. The current literature mostly consists of case reports and case series rather than systematic reviews and meta-analyses, something that confirms the rareness of isolated acute DRUJ dislocations. In addition to other aspects of this type of injury, treatment strategies also remain unclear, with some practitioners advocating for conservative management while others recommending surgical approaches when conservative treatment fails or when persistent instability is present [16].

This systematic review aims to collate the results of all available literature in regard to treatment protocols for acute isolated DRUJ dislocations. By analyzing the evidence from the existing bibliography, this review intends to provide a detailed framework and shed light on the efficacy of various management strategies and their impact on functional recovery.

## 2. Materials and Methods

PubMed, Scopus, and Mendeley databases were used during this review. They were searched from January 2000 to November 2024, using the key terms “distal radioulnar joint dislocation”, “isolated”, and “acute”. Furthermore, a backwards citation search was conducted in the relevant articles to identify any studies that may have been missed in the initial search. The Preferred Reporting Items for Systematic Reviews and Meta-Analyses (PRISMA) 2020 guidelines were followed to ensure the validity of methodology.

The studies included in this systematic review met our specific eligibility criteria. The population of interest was adult patients who sustained traumatic isolated DRUJ dislocations, volar or dorsal, and the diagnosis as well as the management were established within 1 week of dislocation. Our exclusion criteria were patients under 18 years of age, DRUJ dislocations with associated fractures, delayed diagnosis or treatment (beyond 1 week), and open injuries. Due to the uncommon nature of this injury, there is a paucity of literature regarding acute isolated DRUJ dislocations. Consequently, we included mostly case reports and case series where full text manuscripts were available.

The demographics of patients with this type of injury were documented in addition to the type of dislocation, mechanism of injury, dominant hand injury, and the use of additional imaging studies. It was deemed appropriate to also record the missed diagnosis rate at first presentation of the included cases. Of course, the preferred treatment protocol, the follow-up length, and the reported outcomes of each case were described to meet the requirements of this systematic review.

**Figure 1:**
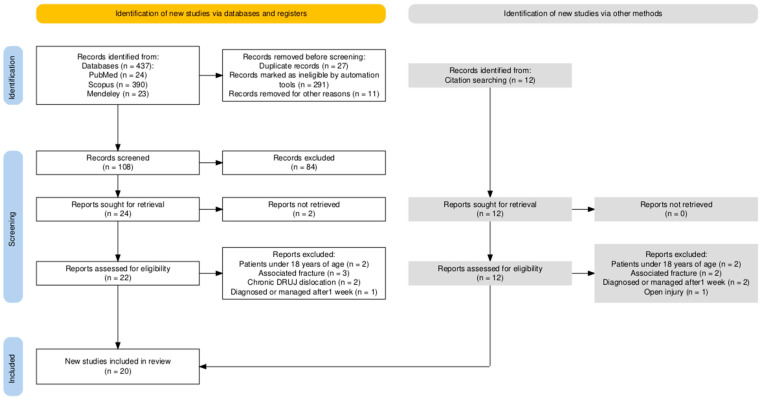
PRISMA 2020 flow diagram for the search protocol used to review treatment of acute isolated DRUJ dislocations. DRUJ: Distal Radioulnar Joint.

Our initial database search attributed 437 papers. The “Adults” and “English” filters were applied to the Scopus search engine, and as a result, 291 papers were excluded automatically. Moreover, all duplicate records (*n* = 27) and articles published before 2000 (n = 11) were manually removed. Further, 12 papers derived from backward citation search were assessed for eligibility. After eliminating the studies that did not match our inclusion criteria, we were left with a total of 20 papers. The process of our literature search according to PRISMA 2020 guidelines is analytically presented in Figure 1.

## 3. Results

In total, 20 studies met our eligibility criteria and were included in the final review [2,4,6,12,14,16,17,18,19,20,21,22,23,24,25,26,27,28,29,30]. Of these 20 studies, 15 studies were purely case reports. There was 1 case series study, and 3 of the 20 studies were case reports combined with literature reviews [6,20,25,30]. The remaining study provided a case series along with a systematic review of the literature on isolated volar dislocations of DRUJ [16]. All the included studies provided information about 23 cases in total. One case described in O’Malley et al.’s case series and systematic review had to be excluded due to delayed treatment with closed reduction of DRUJ dislocation at 3 weeks [16]. As a consequence, this systematic review refers to a total of 22 cases.

Males comprised a major part of the patients sustaining acute isolated DRUJ dislocations. Of the total 22 patients, 20 were males in comparison to only 2 females. There was also a broad spectrum of ages amongst all studies. The mean age was 37.9 years of age, ranging from 20 to 70 years of age.

The type of dislocation was documented in all studies. Overall, 18 of the reported cases sustained a volar dislocation in contrast to 4 cases with dorsal dislocation. All studies also made reports on the mechanism of injury. Falls were the most common cause of injury, as in 9 of the 22 patients led to DRUJ dislocations [4,6,14,17,18,23,27,28,30]. More precisely, 3 patients sustained a fall onto an outstretched hand, 1 patient suffered a fall from a scooter accident, and 1 patient suffered a fall as a football injury [4,6,17,18]. Direct impact force on the wrist was the causing mechanism of injury for 5 patients, and in 1 case this happened during a martial arts competition [6,16,19,25,29]. Rugby injuries occurred in 4 of the patients; 1 patient was injured in a motorcycle accident, 1 patient was injured after punching a wall with a clenched fist, and 1 patient was injured during a friendly grappling match [2,12,16,20,21,24,26]. In 1 case the mechanism of injury was unclear [22]. Sport injuries, including rugby, martial arts, and football injuries, were the cause of traumatic dislocation in 6 patients [12,17,20,24,25,26]. In 15 of the 20 studies, where 16 cases were described, there was documentation of dominant hand injury [2,4,6,14,17,18,19,20,21,22,24,25,26,27,30]. Overall, 9 of the 16 patients sustained a DRUJ dislocation affecting their dominant hand [4,6,14,18,19,21,26,27,30].

The diagnosis of acute isolated DRUJ dislocation was missed at first presentation in 4 of the 22 included cases, which is equivalent to a misdiagnosis rate of 18%. In clinical presentation, all cases expressed pain of variable intensity located on the wrist and some form of restriction in pronation and supination. Occasionally, this movement restriction was present exclusively in one direction. In 6 patients with volar dislocations, the wrist was held in supination, whereas pronation was impossible [6,21,23,24,26,27]. Saadi et al. reported a case with dorsal dislocation, where supination was unrestricted, and pronation was impossible [17]. On the contrary, another case with dorsal dislocation reported by Wassink et al. presented with unrestricted pronation while supination was unfeasible [28]. For 2 patients, there were reports that wrist flexion and extension were maintained, and 3 patients presented also with maintained finger flexion and extension [2,12,14,25,29]. Furthermore, absence of the normal ulnar head prominence dorsally was a characteristic finding in 9 patients, which sustained volar DRUJ dislocations [14,19,20,21,22,24,26,29,30]. All cases presented with intact neurovascular status, except Sreenivasan et al.’s case, where a mildly reduced sensation in the ring and middle fingers was reported on light touch, but no paresthesia was present [12]. The dislocation workup was enhanced by further imaging studies in 13 cases [4,6,12,16,18,19,20,21,22,23,25,30]. Magnetic resonance imaging (MRI) was used in 8 cases, mainly aiming to assess concurrent soft tissue injuries, like TFCC injuries. In one of them, MRI was performed before reduction to confirm diagnosis [6,12,16,18,20,23,25,30]. A computed tomography scan (CT) was also utilized as an accessory imaging technique in 7 cases [4,16,18,19,21,22,23]. In 4 cases, CT scan was performed prior to reduction, in 1 case following a successful reduction, and in 2 cases following an unsuccessful reduction attempt. Both additional imaging tests were used in 2 cases [4,16,18,19,20,21,22,23].

There was a wide diversity regarding treatment strategies. In total, 16 of the 22 patients were managed conservatively with a successful closed reduction followed by different forms of immobilization [6,12,14,16,18,21,22,23,24,25,27,28,29,30]. Various forms of anesthesia were used as well, ranging from conscious sedation to general anesthesia. The mean immobilization period in the conservative treatment group was 4.9 weeks (from 3 to 6 weeks). Operative treatment was chosen for 6 of the 22 patients [2,4,17,19,20,26]. A closed reduction followed by fixation with Kirschner wires (k-wires) was the treatment of 4 patients, while 2 patients underwent an open reduction followed by reattachment of TFCC in one case and dorsal radioulnar ligaments plus k-wire fixation in the other [2,4,17,19,20,26]. A postoperative immobilization period was also necessary with a mean duration of 5.3 weeks (from 4 to 6 weeks). A total of 7 of the 22 patients went through physiotherapy sessions during their rehabilitation [6,14,17,18,19,20]. Interestingly, an initial closed reduction attempt was unsuccessful in 9 patients, and this was the reason that led to surgical treatment in 5 cases [4,12,16,17,19,20,21,26,28]. Overall, 17 of the 20 studies, regarding 18 patients, referred to follow-up length [2,4,14,16,17,18,19,20,21,22,23,25,26,27,28,29,30]. There was a substantial range from 3 weeks to 4 years, with a mean follow-up length of 8.53 months.

There was a various degree of inconsistency concerning the choice of different outcome measures. All studies except 2 documented some form of specific outcome measures [6,24]. Consequently, there are reports about outcomes for 19 patients in total. For 17 of these 19 patients, there was available data about the range of motion (ROM) at follow-up. In total, 14 of 17 patients (82%) achieved full ROM at follow-up. On the other hand, 3 patients (18%) referred to some limitations in ROM, and, more specifically, 1 patient experienced a limited ROM in supination, 1 patient in both supination and pronation, and 1 patient in wrist flexion and extension [19,23,28]. Pain or symptoms were reported in 16 of the 19 patients. Of these, 14 patients (88%) had no complaint about pain or symptoms, while of the remaining 2 (12%), 1 patient reported minimal pain and 1 patient referred pain in forced pronation [21,25]. Patient satisfaction was referred only in 2 cases, where both were satisfied [4,29]. DRUJ stability was assessed in 10 of the 19 patients and in all (100%) of whom DRUJ had no laxity [2,4,14,16,17,18,19,21,25]. Restrictions in work or sport-related activities were evaluated in 8 of the 19 patients, and none of them (0%) had any restrictions [2,4,17,18,19,20,25,29]. X-ray follow-up was performed in 5 of the 19 patients, and in all of them (100%), reduction was maintained successfully [14,19,21,22,25]. In 4 of the 19 patients, the handgrip strength was evaluated and in all of them strength was equal to the non-injured hand [2,4,17,25]. Regarding functional outcomes based on quantitative data, only 1 case report used DASH score, which was 0/100 at follow-up, and in one patient, VAS score was used, which was 3/10 in forced pronation [4,25]. Finally, in only 1 case a recurrent dislocation was described 8 months later, following a new traumatic event [28]. Table S1 summarizes all the available data extracted from the included publications. The accumulative data concerning the outcomes of acute isolated DRUJ dislocation treatment are summarized in Table S2.

## 4. Discussion

Acute isolated DRUJ dislocations constitute a rare clinical entity, which remains underrepresented in the orthopedic literature [4]. Routinely, this injury is accompanied by distal radius and ulnar styloid fractures, or it is combined with more complex injuries, such as Essex–Lopresti or Galeazzi fractures [1,2,31]. The scarcity of this injury explains why the bibliography is mainly comprised of reports from individual cases. By synthesizing data from 22 cases across 20 studies, this systematic review has attempted to elucidate patterns in presentation, diagnosis, treatment strategies, and reported outcomes.

The most common mechanisms of injury involved falls and sports-related trauma, particularly rugby, martial arts, and football. Male predominance (90.9%) and an average age of 37.9 years reflect the demographic profile of individuals at higher risk for such injuries, likely due to their participation in high-impact work or recreational activities. The dominant hand was more frequently affected, potentially indicating a higher risk during falls or protective maneuvers. Isolated dorsal DRUJ are more common than volar [30]. However, our results contradict this established knowledge, as in 18 of the 22 patients, the direction of the dislocation was volar. This discrepancy can be attributed to the small sample size, which may not provide a robust representation of the general trend. In addition, our patient cohort was derived almost exclusively from case reports, where the authors tend to publish more unusual pathologies.

The prompt identification of an isolated DRUJ dislocation requires a high index of suspicion, as it can mimic other soft tissue injuries [4]. Our review suggests a misdiagnosis rate at first presentation as high as 18%, which is noticeably lower than the existing literature reports ranging from 36% to 50% [2,3,16]. We emphasize that the high rate of misdiagnosis may result in delays in the initial management of the dislocation, leading to unfavorable consequences for the patient. The clinical presentation of a traumatic isolated dislocation may vary, depending on the direction of dislocation and the mechanism of injury. The possible loss of normal palpable anatomical landmarks due to local swelling, the often-maintained wrist flexion and extension, the inability to obtain correct lateral X-rays, and the absence of fractures radiologically can make the diagnosis a great challenge [19,27]. The findings in anteroposterior (AP) view differ according to the type of dislocation. Volar dislocations typically result in an overlap of the distal radius and ulna due to the pull of the pronator quadratus, while dorsal dislocations lead to joint widening [27]. Surprisingly, only 10 degrees of pronosupination in the lateral view can result in misleading X-rays [5,6]. In a true lateral view, the volar cortex of the pisiform appears to sit within the interval between the volar cortices of the capitate and distal pole of the scaphoid, ideally within the central third of this interval [6]. Some authors suggest the use of comparative wrist X-rays when the diagnosis of an acute isolated DRUJ dislocation is suspected [2]. Diagnostic accuracy can be augmented by further imaging studies like CT and MRI when X-rays are inconclusive or associated bony and soft tissue injuries need to be assessed [2,30].

To this date, there is no consensus about the most appropriate treatment strategy. The management of acute isolated DRUJ dislocations should long-term focus on the prevention of chronic instability, pain, and osteoarthritic changes of the joint [3]. Most of our included cases were treated successfully with closed reduction followed by an immobilization period. There is heterogenicity in the immobilization medium, which often changed from above-elbow to below-elbow at some point in the treatment protocol. The onset of mobilization, with the use of physiotherapy or not, varied amongst studies, but in the conservative treatment group, it was after a mean of 4.9 weeks. This is slightly lower than the healing time of TFCC (6–8 weeks), which is almost always considered to be injured [14,24]. The sufficient vascularity and healing potential of peripheral TFCC usually leads to a successful non-surgical treatment [2]. The maneuver of closed reduction usually requires some form gentle traction, pronation or supination, and pressure of the ulnar head in a direction dependent on the type of dislocation [24]. Occasionally, closed reduction may be impeded by muscle contraction of the pronator quadratus or soft tissue interposition, such as the articular capsule, TFCC, and ECU [12,14,24,30]. Moreover, isolated DRUJ dislocations may result in an impaction of the ulnar head to the distal radius, an injury equivalent to Hill–Sachs lesions seen after shoulder dislocations [6]. This impaction may not be visible on plain films and can also prevent closed reduction, as it happened with the cases reported by Larrivée et al. and Garrigues et al. [6,12,19,21]. In the majority of the cases included, the level of expertise of the physician that attempted closed reduction is not reported. We suggest that the high rates of initially failed closed reduction attempts may be attributed to insufficient experience in managing this rare type of injury.

Operative treatment appears to also be a considerable management option for a smaller part of patients with acute isolated DRUJ dislocations, as it worked effectively for six of our included cases. Although some authors recommend surgical approach as a routine, our findings suggest that operative management is absolutely indicated in irreducible isolated dislocations and in cases where there is an unacceptable residual instability of the DRUJ following a successful closed reduction [25]. In five of the patients that underwent surgical management, an unsuccessful reduction attempt had preceded. In most cases, the stabilizing structures of the joint are sufficient to maintain stability [25]. If this is not the case, fixation with k-wires is a viable option. In cases of inability to achieve closed reduction, an open reduction is indicated. Postoperatively, an immobilization period is usually necessary, the duration of which is debatable and should be considered by surgeons. The protection of the biomechanical construct created in the operating room may require a longer immobilization and consequently a slower return to work. Darrach’s and Sauvé–Kapandji procedures were not used in any of our included cases, and they might be more suitable for established chronic DRUJ instability [32].

Osteoarthritis induces significant alterations in both the macrostructure and microstructure of cartilage, compromising its mechanical properties [33]. However, there is no clear evidence regarding the severity of osteoarthritis potentially resulting from the specific injury under study, likely due to the short follow-up periods reported in our studies. Specifically, the average follow-up duration is only 8.53 months, limiting the ability to assess long-term outcomes related to osteoarthritis. In addition to osteoarthritis, chronic wrist instability cannot be adequately assessed in the current follow-up of the patients, and future studies with longer follow-up periods (2 or 3 years) could certainly enrich the results.

The functional outcomes of acute traumatic DRUJ dislocation are generally satisfactory, regardless of the method of treatment. In most cases, patients achieve full range of motion of the radiocarpal joint and return to their functional status prior to the injury [24]. Even patients with limited range of motion show relatively small deficits in pronation and supination, as well as in flexion and extension of the radiocarpal joint, although it is not clear whether this limitation significantly affects their daily activities [19,23,28]. It is worth noting that the patients who were asked if there were any restrictions in their work or sports activities all responded negatively [2,4,17,18,19,20,25,29]. We recommend that authors presenting future studies on this specific injury should record whether their patients returned to work functionally, as reliable conclusions can be drawn regarding the cost, depending on the therapeutic approach.

After the completion of treatment (either surgical or conservative), the majority of patients report no pain in the radiocarpal area at the final follow-up. In only two cases is minimal pain reported, but the follow-up periods are relatively short (3 and 14 weeks), which may lead to an unreliable result [25]. We believe that this data, namely the combination of range of motion and pain levels, can support the good functional outcomes of the patients. Additionally, DRUJ stability is an important outcome measure, and encouragingly, this was achieved in all studies that assessed it. The patient reported by Wassink et al. is the only case to our knowledge that refers to a recurrent dislocation after a new traumatic incident [28]. Patient satisfaction is directly related to functional outcomes; however, unfortunately, this parameter has been recorded in only two cases [4,29]. Although these patients were satisfied with their functional outcomes at the end of follow-up, reliable conclusions cannot be drawn between this injury and patient satisfaction.

Acknowledging the limitations of this study is essential for interpreting its findings accurately. There is a paucity of large, comprehensive studies regarding acute isolated DRUJ dislocations. Our review included patients almost entirely from case report studies with no control groups, so selection bias is a limitation that is highly considered. In addition, the total number of patients is small, so it is possible that the studied population is not statistically representative of the general population. The mean follow-up length is also relatively short, and this may not allow for observation of long-term outcomes. The interpretation of outcomes should ideally be based on quantitative data, such as outcome scores, and in the selected cases, such data are lacking. Furthermore, treatment considerations should likely include additional parameters, such as the specific needs of different patient groups. For example, the studied cases did not include any distinct subpopulations, such as elite athletes or low-demand elderly individuals. We suggest that authors working on this specific topic record more parameters in their studies to ensure greater homogeneity between studies.

## 5. Conclusions

This systematic review summarized all the available literature about acute isolated DRUJ dislocation in an attempt to enhance clinician awareness and provide insights about optimal management. This injury pattern is rare and surrounded by diagnostic challenges, mainly derived from subtle clinical presentation and the inability to obtain correct imaging. Conservative treatment, involving closed reduction and immobilization, is successful for most patients, given that the dislocation is promptly diagnosed in the acute phase. Surgical management is reserved for irreducible dislocations or cases with remaining instability after closed reduction. Functional outcomes, in terms of ROM, residual pain, stability, and return to work and sport-related activities, are generally excellent, irrespective of treatment modality. However, this conclusion cannot be drawn with definite safety due to the small number of cases included, the short follow-up length, and the possible selection bias that encompasses case reports. Future research is required, optimally with a prospective design, to focus on standardizing outcome measures, including patient satisfaction, long-term follow-up data, and return-to-function metrics, to provide clearer guidance for clinicians.

## Data Availability

Not applicable.

## Supplementary Materials

The following supporting information can be downloaded at: https://www.mdpi.com/article/10.3390/jcm13247817/s1, Table S1: All the available data as presented in the accepted publications; Table S2: Literature data, including outcomes of acute isolated DRUJ dislocation. * DRUJ: Distal Radioulnar Joint.
